# Treatment of cephalic arch stenosis in dysfunctional arteriovenous fistulas with paclitaxel-coated versus conventional balloon angioplasty

**DOI:** 10.1186/s42155-021-00271-1

**Published:** 2021-11-29

**Authors:** Ren Kwang A. Tng, Ru Yu. Tan, Shereen X. Y. Soon, Suh Chien. Pang, Chieh Suai. Tan, Charyl J. Q. Yap, Apoorva. Gogna, Tze Tec. Chong, Tjun Y. Tang

**Affiliations:** 1 Department of Renal Medicine, Singapore General Hospital, Singapore, Singapore; 2 Department of Vascular Surgery, Singapore General Hospital, Singapore, Singapore; 3 Department of Vascular Interventional Radiology, Singapore General Hospital, Singapore, Singapore; 4 Duke NUS Graduate Medical School, Singapore, Singapore

**Keywords:** Cephalic arch stenosis, Paclitaxel, Angioplasty, Arteriovenous fistula

## Abstract

**Background:**

Treatment of cephalic arch stenosis (CAS) with standard plain old balloon angioplasty (POBA) in dysfunctional arteriovenous fistulas (AVF), is associated with early re-stenosis and higher failure rates compared to other lesions. Paclitaxel-coated balloons (PCB) may improve patency rates. This is a retrospective cohort study. Patients who underwent POBA or PCB for CAS over a 3-year period were included. Outcomes compared were circuit primary patency rates (patency from index procedure to next intervention), circuit primary assisted-patency rates (patency from index procedure to thrombosis), and target lesion (CAS) patency rates (stenosis > 50%) at 3, 6 and 12 months.

**Results:**

Ninety-one patients were included. Sixty-five (71.4%) had POBA, while 26 (28.6%) had PCB angioplasty. There were 62 (68.1%) de-novo lesions. CAS was the only lesion that needed treatment in 24 (26.4%) patients. Circuit primary patency rates for POBA versus PCB groups were 76.2% vs. 60% (*p* = 0.21), 43.5% vs. 36% (*p* = 0.69) and 22% vs. 9.1% (*p* = 0.22) at 3, 6 and 12-months respectively. Circuit assisted-primary patency rates were 93.7% vs. 92% (*p* = 1.00), 87.1% vs. 80% (*p* = 0.51) and 76.3% vs. 81.8% (*p* = 0.77), whilst CAS target lesion intervention-free patency rates were 79.4% vs. 68% (*p* = 0.40), 51.6% vs. 52% (*p* = 1.00) and 33.9% vs. 22.7% (*p* = 0.49) at 3, 6 and 12-months respectively. Estimated mean time to target lesion intervention was 215 ± 183.2 days for POBA and 225 ± 186.6 days for PCB (*p* = 0.20).

**Conclusion:**

Treatment of CAS with PCB did not improve target lesion or circuit patency rates compared to POBA.

## Background

Vascular access, either via fistula (preferred), graft or catheter is a prerequisite to hemodialysis (Lok et al. 2020; Zavacka et al. 2020). However, the access is vulnerable to dysfunction with stenosis developing along the circuit. Cephalic arch stenosis (CAS) occurs commonly in brachiocephalic AVFs, with a reported prevalance of up to 77% (Hammes et al. 2008). Treatment of AVF stenosis with standard percutaneous transluminal angioplasty (PTA) is generally associated with high success and efficacy (Yildiz 2020), along with favourable safety profiles despite the risks of trauma to the vessel wall (Zhang et al. 2020). However, PTA for CAS is associated with higher failure and complication rates and a tendency for early re-stenosis compared to other lesions (Sivananthan et al. 2014).

The definition of the cephalic arch varies across literature, with different papers referring to the central perpendicular portion of the cephalic vein as it traverses the deltopectoral groove and joins the axillary vein (Rajan et al. 2003), or the final arch of the cephalic vein before it joins the axillary vein (Kian and Asif 2008). Bennet further divided the cephalic arch into 4 segments, based on the distance from the arch apex to the cephalic-axillary vein junction (Bennett et al. 2014). The causes of stenosis at the cephalic arch have not been clearly elucidated, with various postulated contributing factors including: increased blood flow and pressures resulting in neotintimal hyperplasia, extrinsic compression by the deltopectoral and claviculopectoral fascia, morphology of the cephalic arch, and the angle of the cephalic-axillary vein junction with increased turbulent flow, and increased number of valves in the cephalic vein resulting in increased turbulent flows (Sivananthan et al. 2014). These factors have contributed to the occurrence and recurrence of the lesion, and dialysis circuit primary patency and assisted-primary patency rates remain less than ideal with plain old balloon angioplasty (POBA) alone. A systematic review of endovascular management of CAS in failing brachiocephalic fistula also demonstrated effective short-term (6- and 12-months) primary patency rates with the use of stent graphs over endovascular modialities such as bare metal stents (D’cruz et al. 2019). However PTA remained necessary to maintain longer term patency of the stent graphs. Hence, there is a need for a longer term treatment solution for the cephalic arch stenosis.

Paclitaxel-coated balloons (PCB) have been used successfully to increase the patency of arteriovenous accesses in several large randomized controlled trials (Lookstein et al. 2020; Trerotola et al. 2018; Swinnen et al. 2018; Irani et al. 2018). However, these studies have looked at AV accesses as a whole, and not specifically at the cephalic arch. PCBs were made available in our centre for the treatment of dysfunctional arteriovenous access recently, and the objective of this retrospective audit was to report the dialysis circuit and CAS target lesion patency rates following DCB angioplasty of CAS compared to POBA.

## Methods

All patients who presented to the institution with dysfunctional AVFs from January 2017 to December 2019 were retrospectively reviewed. A total of 91 unique patients who had treatment for CAS were included in the analysis. Clinical data and procedural reports were obtained from electronic medical records and all patients had a follow-up of at least 12 months following the index procedure. Demographic characteristics were collected. Outcome measures were circuit primary patency rates, defined as patency following index procedure until the next re-intervention within the access circuit, circuit primary assisted-patency rates, defined as the circuit patency following index procedure until the next access thrombosis, and cephalic arch (target lesion) patency rates defined as a stenosis > 50% at the CAS at 3, 6 and 12 months. The mean time to target lesion intervention was also determined with Kaplan-Meier analysis. This quality audit performed with de-identified data was exempted from Institutional Review Board review (CIRB Ref: 2020/2320).

All procedures were performed in a hybrid operating theatre using standard percutaneous techniques under local anaesthesia and/or sedation. An initial fistulogram was obtained. Cephalic arch stenosis was defined as > 50% stenosis at the cephalic arch. Decision to treat the CAS, as well as the type of balloon used, was operator dependent. The CAS was crossed in the usual way and mandatory pre-dilatation of the target lesion with a standard high-pressure non-compliant balloon (Mustang®, Boston Scientific, Marlborough, MA, USA) for 2 min was used, with balloon size matched to the calibre of the adjacent normal vessel. After appropriate lesion effacement (< 30% residual stenosis), the PCB was inserted (sizing 1:1 to pre-dilatation balloon) and inflated for 2 min to a pressure according to the design specifications of the PCB to allow maximal drug transfer to the vessel wall. Balloon length was chosen to be at least 2 cm longer than the area treated during pre-dilatation (1 cm overlap proximal & distal) in order to avoid geographical miss. Post-procedure, all patients received daily aspirin (100 mg) and clopidogrel (75 mg) plus a proton pump inhibitor for 1 month, followed thereafter by single antiplatelet agent therapy.

Continuous variables were summarized as mean ± standard for normally distributed variables, and median (25th percentile, 75th percentile) for variables that were not normally distributed, while categorical variables were reported as frequency counts and percentages. Pearson chi-square test was used to compare categorical variables and student t-test or wilcoxon rank sum test were used for continuous variables. The mean time to target lesion intervention was estimated and compared with Kaplan-Meier analysis. Statistical analyses were conducted using SPSS (IBM, Version 21).

## Results

**Fig. 1:**
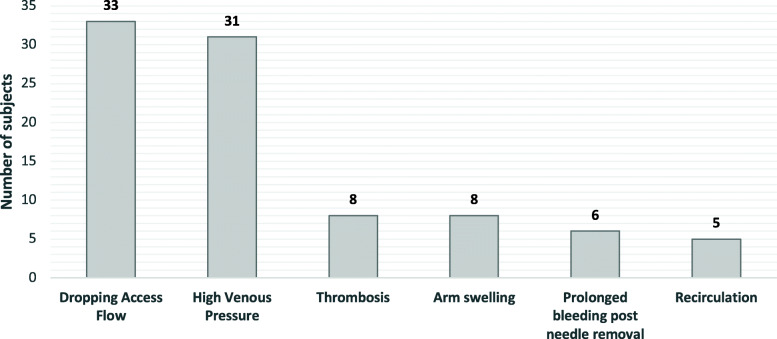
Reasons for re-intervention (index procedure)

Of the 91 patients included, 65 (71.4%) patients were treated with POBA, while 26 (28.6%) had angioplasty with PCB. Majority of the patients were female gender (53.8%), of Chinese ethnicity (68.1%), with mean age of 65.3 ± 10.6 years (Table 1). The type of access was mainly brachiocephalic AVF (94.5%), with a small proportion of radiocephalic AVF (5.5%). The demographics of the 2 groups were similar in terms of their co-morbidities of hypertension, hyperlipidemia, diabetes mellitus, cerebrovascular and cardiovascular diseases (Table 1). The indications for intervention are summarized in Fig. 1. Out of 32 PCBs used in 26 patients, 22 (68.8%) were of Paclitaxel dosing of 2μg/mm^2^, 1 (3.1%) balloon with a dosing of 3μg/mm^2^, 5 (15.6%) with a dosing of 3.5μg/mm^2^, while the Paclitaxel dosing of the 4 (12.5%) remaining PCBs were not stated.

**Table 1:** Patient demographics by treatment type

	Number of subjects (%)			
	Total (*n = 91)*	POBA (*n = 65*)	PCB (*n = 26*)	***p***-value
Mean Age, years (±sd)	65.3 ± 10.6	64.7 ± 10.4	66.6 ± 11.3	.461
Mean BMI, kg/m^2^ (±sd)	26.4 ± 5.1	26.1 ± 4.6	27.0 ± 6.3	.532
Gender				
Female	49 (53.8)	36 (55.4)	13 (50.0)	.816
Male	42 (46.2)	29 (44.6)	13 (50.0)	
Ethnic Group				
Chinese	62 (68.1)	44 (67.7)	18 (69.2)	.552
Malay	25 (27.5)	19 (29.2)	6 (23.1)	
Indian	4 (4.4)	2 (3.1)	2 (7.7)	
Smoker	14 (15.4)	9 (13.8)	5 (19.2)	.532
Co-Morbidities (%)				
Hypertension	90 (98.9)	64 (98.5)	26 (100)	1.00
Hyperlipidemia	77 (84.6)	56 (86.2)	21 (80.8)	.532
Diabetes	67 (73.6)	47 (72.3)	20 (76.9)	.851
Nephropathy	57 (62.6)	38 (58.5)	19 (73.1)	.288
Retinopathy	36 (39.6)	27 (41.5)	9 (34.6)	.709
Neuropathy	12 (13.2)	8 (12.3)	4 (15.4)	.737
Coronary Artery Disease	41 (45.1)	29 (44.6)	12 (46.2)	1.00
Cerebrovascular Accident	9 (9.9)	5 (7.7)	4 (15.4)	.270
Malignancy	8 (8.8)	4 (6.2)	4 (15.4)	.219
Asthma	3 (3.3)	3 (4.6)	0	.555
Access Side				
Left	61 (67.0)	41 (63.1)	20 (76.9)	.307
Right	30 (33.0)	24 (36.9)	6 (23.1)	
Access Type				
Brachiocephalic	86 (94.5)	61 (93.8)	25 (96.2)	1.00
Radiocephalic	5 (5.5)	4 (6.2)	1 (3.8)	
Access Patency				
Recurrent	59 (64.8)	41 (63.1)	18 (69.2)	.755
De novo	32 (35.2)	24 (36.9)	8 (30.8)	
Mean Access Age, months (25th, 75th percentile)	62 (35, 145)	52 (36, 144)	71 (28, 171)	.857
Medical History				
Statin	73 (80.2)	51 (78.5)	22 (84.6)	.708
Beta Blocker	61 (67.0)	44 (67.7)	17 (65.4)	1.00
Antiplatelet	60 (65.9)	42 (64.6)	18 (69.2)	.861
Antidiabetic agents	50 (54.9)	37 (56.9)	13 (50.0)	.714
Warfarin	7 (7.7)	3 (4.6)	4 (15.4)	.100

*POBA* Plain Old Balloon Angioplasty, *PCB* Paclitaxel Coated Balloon

Table 2 summarizes the details of the characteristics of the cephalic arch stenosis while Table 3 summarizes the other lesions treated concurrently during the index procedure. Majority of the CAS was de novo (68.1%). In patients with recurrent CAS, median time from prior intervention was 236 (Interquartile range (IQR) 163, 564) days and was similar between the 2 groups. CAS was the only lesion that needed treatment in 24 (26.4%) patients. There were a total of 106 other treated lesions in the remaining 70 AVFs, with juxta-anastomotic stenosis being the most common lesion (23.7%) followed by mid-cephalic vein (8.1%), cannulation zone (7.6%), central venous system (5.1%), proximal cephalic vein (4%), distal outflow (2%) and proximal basilic vein (0.5%). The severity of CAS was greater in the PCB group with percentrage of stenosis being 83 ± 10.4% compared to 75 ± 12.4% for the POBA group (*p* = 0.001).

**Table 2:** Characteristics of cephalic arch stenosis

	Total (*n = 91)*	POBA (*n = 65*)	PCB (*n = 26*)	***P***-value
**Cephalic arch**				
Single lesion	24 (26.4)	16 (24.6)	8 (30.8)	0.409
Percentage stenosis, %	78 ± 12.3	75 ± 12.4	83 ± 10.4	0.006
Prior stenting, n (%)	6 (6.5)	2 (3.1)	4 (15.4)	0.053
De novo	62 (68.1)	46 (70.8)	16 (61.8)	0.545
Recurrent	29 (31.9)	19 (29.2)	10 (38.5)	
Median time from prior intervention, days (25th, 75th percentile)	236 (163, 564)	236 (174, 608)	268 (98, 452)	0.463

*POBA* Plain Old Balloon Angioplasty, *PCB* Paclitaxel Coated Balloon

**Table 3:** Distribution of other lesions treated during the index procedure

	Total (*n = 106)*	POBA (*n = 88*)	PCB (*n = 18*)
**Other treated lesions**			
Juxta-anastomotic Segment	47 (44.3)	43 (48.9)	4 (22.2)
Mid-Cephalic	16 (15.1)	12 (13.6)	4 (22.2)
Cannulation zone	15 (14.2)	13 (14.7)	2 (11.1)
Central Venous System	15 (14.2)	14 (15.9)	1 (5.6)
Proximal Cephalic	8 (7.5)	5 (5.7)	3 (16.7)
Distal outflow	4 (3.8)	1 (1.1)	3 (16.7)
Proximal Basilic	1 (0.9)	0	1 (5.6)

*POBA* Plain Old Balloon Angioplasty, *PCB* Paclitaxel Coated Balloon

**Fig. 2:**
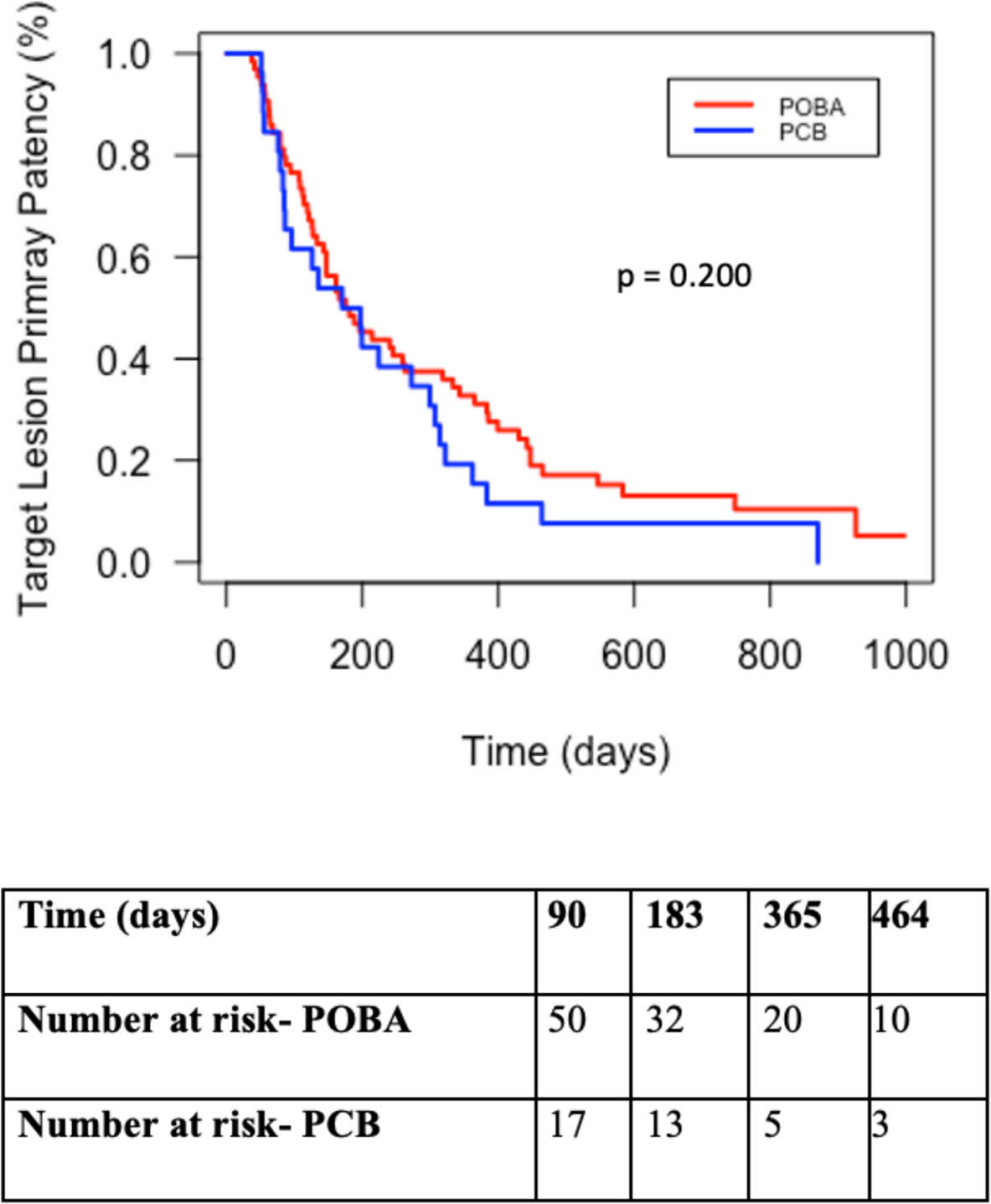
Kaplan-Meier estimates for Target Lesion Primary Patency

There were 6 deaths and 5 abandoned AVFs during the 12 month audit period. The circuit primary patency rates for POBA versus PCB groups were 76.2% vs. 60% (*p* = 0.21), 43.5% vs. 36% (*p* = 0.69) and 22% vs. 9.1% (*p* = 0.22) at 3, 6 and 12-months, respectively. The circuit assisted primary patency rates were 93.7% vs. 92% (*p* = 1.00), 87.1% vs. 80% (*p* = 0.51) and 76.3% vs. 81.8% (*p* = 0.77) while target lesion intervention free patency rates were 79.4% vs. 68% (*p* = 0.40), 51.6% vs. 52% (p = 1.00) and 33.9% vs. 22.7% (*p* = 0.49) at 3, 6, and 12-months, respectively (Table 4). The mean time to target lesion intervention was estimated to be 225 ± 186.6 days for POBA and 215 ± 183.2 days for PCB (*p* = 0.300) (Fig. 2).

**Table 4:** Circuit and target lesion (CAS) patency rates at 3,6, and 12 months

	All	POBA	PCB	***P***-value
**3-month outcomes**				
N	88	63	25	
Circuit primary patency, n (%)	63 (71.6)	46 (76.2)	15 (60)	0.209
Circuit primary assisted patency, n (%)	82 (93.2)	59 (93.7)	23 (92)	1.000
Target lesion patency, n (%)	67 (76.1)	50 (79.4)	17 (68)	0.395
**6-month outcomes**				
N	87	62	25	
Circuit primary patency, n (%)	36 (41.4)	27 (43.5)	9 (36)	0.685
Circuit primary assisted patency, n (%)	74 (85.1)	54 (87.1)	20 (80)	0.508
Target lesion patency, n (%)	45 (51.7)	32 (51.6)	13 (52)	1.000
**12-month outcomes**				
N	81	59	22	
Circuit primary patency, n (%)	15 (18.5)	13 (22)	2 (9.1)	0.219
Circuit primary assisted patency, n (%)	63 (77.8)	45 (76.3)	18 (81.8)	0.767
Target lesion patency, n (%)	25 (30.9)	20 (33.9)	5 (22.7)	0.485

*POBA* Plain Old Balloon Angioplasty, *PCB* Paclitaxel Coated Balloon

## Discussion

In this retrospective analysis, the overall target lesion patency rates of CAS following PTA at 3, 6 and 12-months were 76.1%, 51.7% and 30.9%, respectively, which were comparable with the reported rates in literature (D’cruz et al., 2018). The target lesion patency rates following PTA with PCB at 3, 6 and 12-months were 68%, 52% and 22.7% respectively, with no significant differences compared to treatment with POBA. Mean estimated cephalic arch primary patency rates were also similar for the 2 groups.

While the superiority of PCB over POBA in preventing arteriovenous access re-stenosis has been demonstrated in several large scale randomized controlled trials in recent years, these studies have generally looked at arteriovenous accesses, and not specifically at the cephalic arch (Lookstein et al. 2020; Trerotola et al. 2018; Swinnen et al. 2018; Irani et al. 2018). The pathophysiology of development of stenosis differs across different anatomical sites in AVFs and may influence the treatment outcomes of PTA. This audit specifically compared PCB vs. POBA in the treatment of cephalic arch lesions and our findings failed to demonstrate a significant difference in terms of circuit primary and assisted patency rates and target lesion patency rates. This is likely due to the etiology of CAS being multifactorial in nature, with the anti-proliferative properties of PCB only addressing one of the many factors involved, and hence may not be superior to POBA alone. In addition, the structural properties of the cephalic arch will not be altered by the PCB, with the cephalic arch being compressed by surrounding rigid structures of the deltopectoral and claviculopectoral fascia causing haemodynamically significant stenosis which is susceptible to recoil post-PTA. Another reason contributing to the lack of difference between PCB vs. POBA for our audit could be due to a difference in Paclitaxel dosing of the PCB, with the majority of PCB used in our audit being of a dose of 2μg/mm^2^ compared to other studies with higher dosing of 3.5μg/mm2 (Lookstein et al. 2020; Swinnen et al. 2018; Irani et al. 2018).

Several studies have explored different technologies for prolonging the cephalic arch patency rates. In a small retrospective study including 17 patients with CAS, the use of cutting balloon angioplasty showed 3, 6, and 12-month patency rates of 94%, 81% and 38% respectively (Heerwagen et al. 2010). Although the patency rates appeared better than the reported rates in our audit and existing literature, the study did not directly compare cutting balloon angioplasty with POBA.

Bare metal stents serve as a metallic scaffold that preserve luminal gain following PTA and was thought to be able to prevent recoil and re-stenosis caused by extrinsic compression. However, results on the use of bare metal stents to treat CAS have been variable (D’cruz et al., 2018). Theoretically, in-stent re-stenosis can still occur due to cellular proliferation through the bare stent fenestration. Hence, deploying a stent without combating neointimal hyperplasia may not prolong the patency rates of the cephalic arch.

The stent graft, a type of covered vascular stent to impede neointimal hyperplasia and tissue in-growth, may therefore have the best potential to maintain the patency of the cephalic arch. Although only studied in small populations, the use of stent grafts have been encouraging with positive results reported. Rajan et al. reported target lesion patency at 3, 6 and 12-month of 100%, 100%, 29% respectively for stent graft vs. 60%, 0, 0 for PTA (Rajan and Falk 2015). Shemesh et al. compared bare metal stents to stent grafts for the treatment of CAS, and showed superior primary access patency rates of 39% vs. 82% at 3-months, and 0 vs. 32% at 12-months. It was however surprising to note that the 12-month CAS following stent graft deployment was similar to the reported rates of POBA in the current audit and several other studies (D’cruz et al., 2018; Shemesh et al. 2008). There have been increasing evidence showing that neointimal hyperplasia can occur at stent edges at a later stage, limiting flow and resulting in patency loss. As such, a combination of stent or stent graft with PCB may be the future direction for the treatment of CAS. In a prospective proof of concept study of 8 patients, a combination of helical stent and PCB have been shown to result in primary patency rates of 83.3% at 1-year (Tang et al. 2020). Although the stents used were bare metal stents, the positive results from this small study suggest that stent or stent graft placement in the cephalic arch with PCB treatment in-stent or along the stent edges may prevent neointimal hyperplasia and help to maintain long-term patency for CAS.

Besides endovascular techniques, acute restoration of AVF function and CAS treatment with surgical management has also been described. Davies et al. demonstrated superiority in patency rates of cephalic arch transposition or bypass compared to PTA or bare metal stent placement (Davies et al. 2017). However, the results were confounded by the fact that surgical interventions were offered only to younger and healthier patients. The risks of open surgey may outweigh its benefit in older patients with higher cardiac risk compared to endovascular treatment.

Our audit is limited by several factors: being a single centre audit, the findings may not be generalizable to other patient populations. Due to the inherent limitations of a retrospective audit, the results may have potential confounders. Furthermore, selection bias could not be excluded. Specifically, the group of patients who received PCB had a greater severity of CAS in terms of percentage of stenosis compared to POBA, which may have contributed to the failure to achieve patency improvement with PCB. However, recurrent and de novo stenosis, median time from prior intervention and previous stenting were similar (Table 2). In addition, due to the small sample size of the audit, a type 2 error cannot be excluded. Nonetheless, it is a unique study reporting PCB use solely in one location i.e. the cephalic arch and comparing to a POBA group performed over a similar period of time and by the same operators.

## Conclusions

In conclusion, treatment of CAS with PCB did not improve target lesion or access patency rates compared to POBA. Mechanical scaffolding is required in this unique location of the AVF circuit.

## Data Availability

The datasets generated and/or analysed during the current study are not publicly available due to the Singhealth Centralised Instituitional Review Board requirements but are avaialble from the corresponding author on reasonable request.

## Abbreviations

CAS: Cephalic arch stenosis
PTA: Percutaneous transluminal angioplasty
POBA: Plain old balloon angioplasty
PCB: Paclitaxel-coated balloons
